# Leptin and Leptin Resistance in the Pathogenesis of Obstructive Sleep Apnea: A Possible Link to Oxidative Stress and Cardiovascular Complications

**DOI:** 10.1155/2018/5137947

**Published:** 2018-02-20

**Authors:** Slava Berger, Vsevolod Y. Polotsky

**Affiliations:** Division of Pulmonary and Critical Care and Sleep Medicine, Department of Medicine, School of Medicine, Johns Hopkins University, Baltimore, MD, USA

## Abstract

Obesity-related sleep breathing disorders such as obstructive sleep apnea (OSA) and obesity hypoventilation syndrome (OHS) cause intermittent hypoxia (IH) during sleep, a powerful trigger of oxidative stress. Obesity also leads to dramatic increases in circulating levels of leptin, a hormone produced in adipose tissue. Leptin acts in the hypothalamus to suppress food intake and increase metabolic rate. However, obese individuals are resistant to metabolic effects of leptin. Leptin also activates the sympathetic nervous system without any evidence of resistance, possibly because these effects occur peripherally without a need to penetrate the blood-brain barrier. IH is a potent stimulator of leptin expression and release from adipose tissue. Hyperleptinemia and leptin resistance may upregulate generation of reactive oxygen species, increasing oxidative stress and promoting inflammation. The current review summarizes recent data on a possible link between leptin and oxidative stress in the pathogenesis of sleep breathing disorders.

## 1. Introduction

Obstructive sleep apnea (OSA) is characterized by recurrent upper airway obstructions caused by a loss of pharyngeal muscle tone during sleep. OSA is highly associated with obesity and cardiovascular and metabolic complications, increasing cardiovascular morbidity and mortality. Repetitive upper airway obstructions during sleep lead to fluctuations in blood oxygen levels. These recurrent cycles of hypoxemia and reoxygenation are termed intermittent hypoxia (IH), the most prominent feature of OSA that may contribute to increased production of reactive oxygen species (ROS) and oxidative stress [1]. Sympathetic nerve activation (SNA), another major outcome of the apnea/hypopnea events that contributes to OSA-related comorbidities, is caused by IH and oxidative stress as well as by recurrent arousals from sleep [2–4]. As a result of IH, redox balance in subjects with OSA is altered toward oxidant-producing systems, leading to oxidative stress. Multiple studies observed the presence of oxidative stress in patients with OSA [5–7].

Leptin, also known as a satiety hormone, is a master mediator of food intake and body energy balance. Leptin is produced by adipose tissue and plays multiple regulatory roles in metabolism, immunity, and inflammation [8]. Also, it was shown that leptin is a potent ventilation stimulant acting on central respiratory control nuclei. Leptin exerts its intracellular effects through a long isoform of leptin receptor ObRb. ObRb is found in almost all tissues, and it is expressed at high levels in the brain [9]. Leptin levels in plasma are highly associated with BMI and a degree of adiposity. Therefore, circulating leptin levels are markedly elevated in obese subjects. However, the central satiety effects of leptin are abrogated in obesity. Leptin resistance is defined as a failure of high-circulating levels of leptin to decrease hunger and promote energy expenditure [8]. OSA and IH, powerful triggers of oxidative stress, increase peripheral leptin levels and also induce leptin resistance [10]. Of note, leptin resistance may be implicated in the pathogenesis of OSA through impaired regulation of upper airway patency and diaphragmatic control [11].

Accumulating evidence points to the negative effects of leptin resistance. Both oxidative stress and hyperleptinemia play a role in OSA and IH-associated cardiovascular and metabolic morbidity and mortality. However, the interaction between leptin and oxidative stress in OSA has not been previously systematically examined. The purpose of this paper is to review currently available evidence on these relationships and elucidate the role of leptin and oxidative stress in cardio-metabolic complications of OSA.

## 2. Burden of OSA

Obstructive sleep apnea (OSA) is a major health problem that is characterized by recurrent collapse of the upper airway during sleep leading to intermittent inspiratory flow limitation and cessation of airflow, which results in loud snoring, IH, and hypercapnia during sleep, frequent arousals, and daytime sleepiness [12]. The recurrent upper airway obstructions with a partial or complete occlusion of the upper airway during sleep are termed hypopnea and apnea, respectively [13]. OSA is a major cause of morbidity and mortality in Western society [14–16], which contributes significantly to the development and progression of neurocognitive, metabolic, cardiovascular, and oncologic diseases [17–19]. The prevalence of OSA is 24–27% in middle-aged men, 40–45% in older men, 9% in middle-aged women, and 25–30% in older women, but it exceeds 50% in obese individuals [16, 20]. However, the population-based OSA prevalence may be underestimated because a sleep study is required for the diagnosis, while many asymptomatic and minimally symptomatic patients do not seek medical attention.

## 3. Leptin

First identified by Coleman in 1973, as a circulating factor involved in regulation of body weight, leptin was eventually discovered in 1994 by the group led by Zhang [21]. Leptin is a key regulator of metabolism and body weight that is produced predominantly by adipocytes and, in physiological conditions, is released into the circulation in proportion to body adiposity [22, 23]. Obese subjects usually have high levels of circulating leptin, which is considered a proinflammatory adipokine contributing to the low-grade chronic inflammation in obesity.

In addition to metabolic regulation, leptin may elicit a variety of effects by signaling either centrally or peripherally within a target tissue. On the periphery, leptin has been involved in the regulation of various processes including modulation of inflammatory and immune functions [24], cardiovascular regulation [25], glucose metabolism and insulin secretion [26], reproductive function [27], bone formation [28], and fatty acid metabolism [29]. Leptin action can be mediated by both its endocrine effects (i.e., circulating leptin released from adipocytes) and paracrine effects (produced in situ), since leptin expression has been demonstrated in the bone, mammary gland [30], stomach [31], placenta [32], lungs [33], and carotid body glomus cells [34]. In addition, leptin can also cross the blood-brain barrier through a saturable unidirectional transport system [35], providing the brain with an afferent signal proportional to body fat levels. Leptin exerts its effects through binding to leptin receptor that has several isoforms including isoforms with short and long cytoplasmic domains as well as a soluble isoform. The ObRb isoform that has the longest intracellular domain is found in almost all tissues, and it is the only isoform that can activate the intracellular leptin signaling cascade [9]. ObRb is expressed at high levels in areas of the brain that are involved in the regulation of feeding and energy expenditure. For example, ObRb is expressed in the hypothalamus; in nuclei that are important for metabolic regulation, such as the arcuate nucleus (ARC), ventromedial nucleus of the hypothalamus (VMH), and dorsomedial nucleus of the hypothalamus; and in the lateral hypothalamic area [36]. In addition, ObRb immunoreactivity was demonstrated in the nucleus tract solitaries (NTS), hypoglossal nucleus (12N), and dorsal nucleus of the vagus nerve (DNV) [37]. These data indicate that leptin may have a direct effect on hypoglossal motor neurons maintaining breathing through genioglossal muscle patency.

Despite such unusually high levels of circulating leptin, it appears that obese individuals are unresponsive to its central metabolic and respiratory effects. While in lean subjects leptin tends to circulate at low levels of approximately 5–10 ng/ml [38], it was shown that circulating leptin levels can increase to very high levels of 98 ng/ml after body fat mass reaches 30% [39]. Leptin exerts its satiety effects in the CNS by inducing various neural pathways in the hypothalamus [40]. However, in obese subjects, the central satiety effect of leptin is abrogated [8]. This phenomenon is called leptin resistance and is defined as a failure of high-circulating levels of leptin to decrease hunger and promote energy expenditure. At least three possible mechanisms were proposed to mediate leptin resistance, including a failure of circulating leptin to reach its targets in the brain as a result of the limited permeability of the blood-brain barrier; a downregulation of the ObRb on the cell surface; and/or an inhibition of the leptin-ObRb signaling pathway [41]. In addition, data from animal studies show that diet-induced inflammatory response in the hypothalamus can lead to central leptin resistance and obesity [42]. Leptin resistance may be selective for the central nervous system, and the peripheral actions of leptin can escape leptin resistance and exert its effects on SNA and blood pressure [43–45]. Although novel hormonal treatment, such as amylin, and bariatric surgery are considered, there is no accepted medical treatment for leptin resistance [46–49].

## 4. Leptin Control of Ventilation and Effects of Leptin on the Upper Airway

Accumulating evidence in recent years indicates that leptin can be implicated in the pathogenesis of OSA through central regulation of upper airway patency and diaphragmatic control. Experimental data from animal models show that genetic forms of leptin deficiency and leptin resistance in mice are associated with a higher occurrence of flow-limited breathing and pharyngeal collapsibility [50, 51]. Expression of leptin receptor ObRb in the hypoglossal nucleus innervating GG muscles further indicates a possible direct role of leptin in OSA [37].

Leptin is also involved in the pathogenesis of obesity-associated hypoventilation. Leptin-deficient obese (*ob/ob*) mice exhibit hypoventilation and hypercapnia, which were normalized by leptin replacement therapy. This attenuation in hypercapnic ventilatory sensitivity in *ob/ob* mice was associated with lower tidal volumes compared to weight-matched wild-type mice [52]. Positive effects of leptin replacement on minute ventilation and tidal volumes during flow-limited breathing in *ob/ob* mice were also previously demonstrated by several studies, indicating that leptin treatment may have a potential to treat both OSA and obesity hypoventilation [50, 53]. In addition, genioglossal muscle activity was increased after the leptin treatment, suggesting that leptin modulates neuromuscular response to upper airway resistance [53]. Moreover, intracerebrovascular administration of leptin reversed upper airway obstruction during sleep in leptin-deficient *ob/ob* mice, and this effect was localized in the dorsomedial hypothalamus [51]. In humans, higher leptin levels were associated with increased minute ventilation and reduced pharyngeal collapsibility in a model of experimental upper airway obstruction in women [54]. However, in another study, very high levels of plasma leptin were associated with obesity hypoventilation [55]. Thus, both relative leptin deficiency and leptin resistance may be associated with sleep-disordered breathing in humans.

## 5. OSA and Hyperleptinemia

Elevated plasma leptin levels or hyperleptinemia is associated with various health conditions including hypertension, pulmonary inflammation, and low-grade systemic inflammation and metabolic dysfunction in obese humans. Hyperleptinemia is a component of metabolic syndrome, and recently, it was shown that elevated leptin is a cardiovascular risk factor in metabolically healthy obese subjects [56].

Leptin gene expression and secretion are influenced by many factors. Although the main determinant of leptin levels in the circulation is the amount of white adipose tissue (WAT), other factors such as systemic and tissue hypoxia may influence leptin expression and secretion [10, 57]. Indeed, a growing body of evidence suggests that circulating leptin is increased in response to IH. In fact, clinical data from human studies have shown that subjects with OSA have elevated circulating levels of leptin when compared to controls [10]. Furthermore, increased leptin levels were positively associated with the severity of sleep apnea measured by AHI and oxygen saturation below 90% time [58]. Importantly, the higher leptin levels in OSA were independent of BMI or waist circumference [58]. Yet several studies have reported that markers of adiposity such as fat skin fold, body weight, and BMI are better predictors of hyperleptinemia in OSA rather than AHI [59]. Altogether together, these data indicate that leptin levels are elevated in patients with OSA.

The relationship between hyperleptinemia and OSA was further investigated by interventional studies measuring leptin levels after OSA treatment with continuous positive airway pressure (CPAP). Investigators reported that leptin levels begin to decrease after just a few days of CPAP and remain decreased after a long-term CPAP therapy. It was also shown that a CPAP therapy-induced reduction in circulating leptin levels is not dependent on BMI [60, 61]. Furthermore, a decrease in AHI was found to be an independent predictor of reduced leptin after 6 months of CPAP treatment. Notably, reductions in circulating leptin levels were not restricted to the CPAP therapy only. It was reported that that effective surgical intervention with uvulopalatopharyngoplasty also reduced leptin in subjects with severe OSA [62]. However, several studies reported CPAP therapy does not affect circulating leptin levels in OSA. For instance, a recent study showed that leptin levels and lipid profile of overweight subjects with and without OSA were not different, and that CPAP treatment did not significantly change the BMI, waist and neck circumference, or leptin levels in patients with OSA [63]. Studies on leptin role in children with OSA also demonstrate conflicting results [64–67]. Of note, a recent study conducted on 164 overweight and obese children demonstrated no significant differences in leptin levels between subjects with or without OSA. Correlations between leptin and the oxygen desaturation index were abolished after correction for adiposity [68]. Similar results were also demonstrated in another study suggesting that serum leptin levels are affected by adiposity but not by OSAS severity among children with habitual snoring [69]. These data indicate that efficient OSA therapy can reduce leptin levels in adult patients.

Evidence from animal studies suggests that IH can be a possible mechanism that mediates hyperleptinemia in OSA. For instance, lean mice exposed to 5 days of IH showed an increase in serum leptin levels as compared to mice kept in normoxia [70], indicating that IH may play a role in leptin upregulation and secretion. In fact, it was also reported that hypoxia upregulates the leptin gene promoter activity, mRNA expression, and leptin secretion from cultured adipocytes [71]. Given that leptin is upregulated by hypoxia and that hyperleptinemia may lead to cardiovascular complications, it is conceivable that high leptin levels contribute to cardiovascular complications of OSA. In lean mice, all types of hypoxia induced insulin resistance and increased circulating leptin levels, but only frequent IH (60 times/h) increased lipid peroxidation and TNF-*α* production by adipose tissue [72]. However, two weeks' exposure to IH did not increase serum leptin levels in both lean and obese Zucker rats [73], whereas leptin was upregulated by 2–5 weeks of IH in nonobese or diet-induced obese rats [74, 75]. Such discrepancy in the results indicates that that hyperleptinemia in OSA might be modulated by severity and duration of IH and its interactions with an obese phenotype.

Mechanisms of detrimental metabolic effects of leptin and leptin resistance during IH were addressed in several studies. In wild-type and leptin-resistant rats, IH induced deleterious effects on metabolism and elevated plasma leptin levels through phosphorylation of signal transducer and activator of transcription 3 (STAT3) and expression of proopiomelanocortin (POMC) in arcuate nucleus of the hypothalamus [76]. Activation of the sympathetic nervous system and the renin-angiotensin axis in IH can also stimulate the production and secretion of leptin [77]. Vice versa, leptin is a potent stimulator of the sympathetic nervous system (SNS), and hyperleptinemia may contribute to hypertension in obese humans [43, 44] and rodents [45, 78]. Several studies showed that plasma leptin is elevated in hypertensive patients and it was positively correlated with the development of hypertension in humans [79]. It was also demonstrated that infusions of leptin increase sympathetic activity in humans [80]. In addition, animal study showed that skinny and obese mice overexpressing leptin demonstrated elevation of blood pressure, suggesting that leptin plays a role in the pathogenesis of obesity-related hypertension [81]. Overall, current data show that circulating leptin levels are increased in IH and suggest that hyperleptinemia may be implicated in adverse effects of OSA, such as increased oxidative stress and hypertension.

## 6. Leptin and Oxidative Stress

Growing evidence in recent years indicates that leptin-mediated increase of ROS production and oxidative stress can be one of potential mechanisms that link OSA to an increased risk of cardiovascular morbidity and mortality [82, 83]. Oxidative stress has been implicated in the pathogenesis of aging and multiple diseases including aging; cardiovascular, pulmonary, gastrointestinal, and metabolic diseases, including obesity; and cancer [84–86]. First coined by Sies in 1985, oxidative stress represents a disturbance in balance between reactive oxygen species (ROS) and antioxidants in the cellular redox system [87]. ROS is a common term for chemically reactive chemical species that contain oxygen.

Accumulating evidence from multiple studies links leptin to oxidative stress. It was shown that leptin can induce ROS formation in phagocytic [88, 89] as well as nonphagocytic cells [90–93]. Also, it was reported that high leptin levels may induce ROS generation mainly due to an activation of NADPH oxidase [82, 94, 95]. However, it was also shown that leptin replacement therapy significantly downregulated expression of NADPH oxidase in adipose tissue of leptin-deficient *ob/ob* mice [96], indicating a protective role of leptin at homeostatic levels. Moreover, leptin production was increased by overexpression of endogenous antioxidant enzyme, catalase that catabolizes hydrogen peroxide produced by the dismutation of superoxide, and it was correlated with improved energy expenditure and decreased inflammatory and oxidative stress markers in *ob/ob* mice [97]. The relationship between leptin and oxidative stress has been recently investigated in a mouse model of oxidative stress, demonstrating that increased Nrf2 signaling suppressed oxidative stress and improved leptin resistance in the hypothalamus [98]. In addition, a role of leptin in the establishment of adequate redox system was recently demonstrated by administration of leptin antagonist during neonatal leptin surge, resulting in a lower activity of several antioxidant enzymes in the spleen and proinflammatory profile of cytokines in leukocytes [99]. All in all, these data indicate that leptin plays an important role in the development and regulation of the redox system and that elevated leptin levels, that is, hyperleptinemia, may induce oxidative stress and promote inflammation.

## 7. Interactions between Leptin and Oxidative Stress in OSA

Obesity has been associated with oxidative stress and increased lipid peroxidation, protein carbonylation, and oxidative changes in nucleic acids [100]. Imbalance between ROS and antioxidant systems in obesity was found in both mitochondria and cytoplasm. Multiple studies indicate that OSA and IH interact with obesity-enhancing oxidative stress and oxidative injury in different tissues [101, 102]. Oxidative injury was implicated in multiple complications of obesity and OSA, including multiple cognitive, cardiovascular, endocrine, and oncogenic complications [103, 104]. In parallel, leptin resistance is one of the important pathogenic factors of obesity and OSA [105]. As discussed above, hyperleptinemia and leptin resistance (1) induce oxidative stress in different organs and tissues and (2) lead to adverse outcomes which are remarkably similar to pathogenic sequelae of oxidative stress. Nevertheless, a direct causal link between leptin resistance and oxidative tissue injury in OSA is yet to be demonstrated. Future investigations in this direction will not only improve our understanding of OSA pathogenesis but may also lead to novel therapies.

## 8. Summary

**Figure 1:**
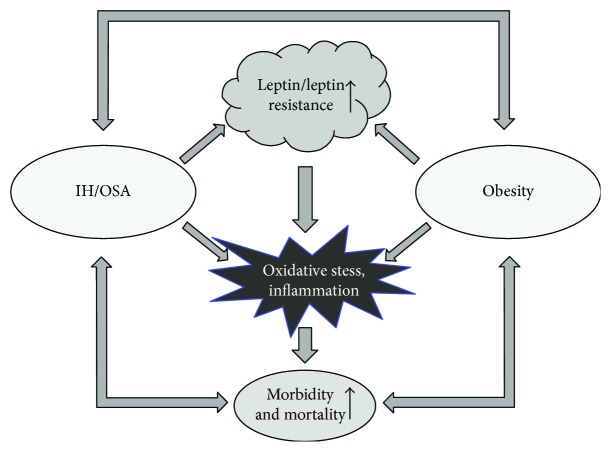
Leptin and oxidative stress may increase morbidity and mortality in obesity and sleep-disordered breathing. Obesity leads to dramatic increases in circulating leptin levels and leptin resistance, while IH/OSA can further augment leptin release from adipose tissue. Hyperleptinemia is associated with increased oxidative stress and can lead to increased cardiovascular risk. IH: intermittent hypoxia; OSA: obstructive sleep apnea.

In summary, IH-induced hyperleptinemia in OSA patients may play a major role in the pathogenesis of sleep apnea. Leptin is a respiratory stimulant, but higher circulating levels of leptin in obese OSA patients fail to modulate the respiratory drive, because of resistance to central effects of leptin. Leptin resistance per se can contribute to development of respiratory sleep disorders. In contrast, the proinflammatory actions of leptin and resulting oxidative tissue injury on the periphery escape central leptin resistance and exert its deleterious effects on SNA, hypertension, and cardiovascular neurologic and metabolic complications in OSA. All of the above suggest that leptin resistance is a target for treatment of sleep breathing disorders including OSA and obesity hypoventilation syndrome (OHS). These findings are illustrated in Figure 1.
